# Non-invasive duo positive airway pressure ventilation versus nasal continuous positive airway pressure in preterm infants with respiratory distress syndrome: a randomized controlled trial

**DOI:** 10.1186/s12887-021-02741-w

**Published:** 2021-07-06

**Authors:** Arash Malakian, Mohammad Reza Aramesh, Mina Agahin, Masoud Dehdashtian

**Affiliations:** 1 Department of Pediatrics, Imam Khomeini Hospital, Ahvaz Jundishapur University of Medical sciences, Ahvaz, Iran; 2 Department of Paediatrics, Ahvaz Jundishapur University of Medical Sciences, Ahvaz, Iran

**Keywords:** Duo positive airway pressure, Nasal continuous positive airway pressure, Preterm infants, Respiratory distress syndrome

## Abstract

**Background:**

The most common cause of respiratory failure in premature infants is respiratory distress syndrome. Historically, respiratory distress syndrome has been treated by intratracheal surfactant injection followed by mechanical ventilation. In view of the risk of pulmonary injury associated with mechanical ventilation and subsequent chronic pulmonary lung disease, less invasive treatment modalities have been suggested to reduce pulmonary complications.

**Methods:**

148 neonates (with gestational age of 28 to 34 weeks) with respiratory distress syndrome admitted to Imam Khomeini Hospital in Ahwaz in 2018 were enrolled in this clinical trial study. 74 neonates were assigned to duo positive airway pressure (NDUOPAP) group and 74 neonates to nasal continuous positive airway pressure (NCPAP) group. The primary outcome in this study was failure of N-DUOPAP and NCPAP treatments within the first 72 h after birth and secondary outcomes included treatment complications.

**Results:**

there was not significant difference between DUOPAP (4.1 %) and NCPAP (8.1 %) in treatment failure at the first 72 h of birth (*p* = 0.494), but non-invasive ventilation time was less in the DUOPAP group (*p* = 0.004). There were not significant differences in the frequency of patent ductus arteriosus (PDA), pneumothorax, intraventricular hemorrhage (IVH) and bronchopulmonary dysplasia (BPD), apnea and mortality between the two groups. Need for repeated doses of surfactant (*p* = 0.042) in the NDUOPAP group was significantly lower than that of the NCPAP group. The duration of oxygen therapy in the NDUOPAP group was significantly lower than that of the NCPAP group (*p* = 0.034). Also, the duration of hospitalization in the NDUOPAP group was shorter than that of the NCPAP group (*p* = 0.002).

**Conclusions:**

In the present study, DUOPAP compared to NCPAP did not reduce the need for mechanical ventilation during the first 72 h of birth, but the duration of non-invasive ventilation and oxygen demand, the need for multiple doses of surfactant and length of stay in the DUOPAP group were less than those in the CPAP group.

**Trial registration:**

IRCT20180821040847N1, Approved on 2018-09-10.

## Background

Respiratory insufficiency is a common problem in term infants and preterm neonates in neonatal intensive care units. In premature infants, the most common cause of respiratory failure is respiratory distress syndrome (RDS) [1]. RDS remains the leading cause of adverse events and mortality in premature infants, affecting approximately 26% of infants born between 32 and 34 weeks of gestation [2].

Historically RDS has been treated by injection of surfactant into the trachea followed by mechanical ventilation. Because of the risk of pulmonary injury associated with mechanical ventilation, followed by the development of chronic lung disease and other complications including subglottic stenosis and pneumonia, less invasive therapies have been proposed to reduce pulmonary complications [3].

In recent years, studies have focused on non-invasive ventilation techniques to reduce the need for mechanical ventilation and its associated pulmonary complications [4]. There are currently a number of non-invasive respiratory care options for preterm infants, including nasal continuous positive airway pressure (NCPAP), nasal intermittent positive ventilation (NIPPV), nasal high frequency oscillation (NHFO) and high flow nasal cannula (HFNC) [5].

One of the common clinical strategies is the use of NCPAP, which has been shown to be effective in reducing ventilation through endotracheal tube and chronic pulmonary disease in very preterm infants [6, 7]. However, in randomized clinical trials, some patients undergoing NCPAP still required intubation due to worsening of patients’ clinical status [8, 9], because NCPAP does not necessarily improve alveolar ventilation or CO_2_ elimination [10].

Currently, NCPAP is the standard treatment for respiratory distress syndrome (RDS) [11]. Duo positive airway pressure (DUOPAP) is a new respiratory support mode consisting of a combination of two CPAP levels. In fact, DUOPAP mode is same as bilevel positive airway pressure (BIPAP). In the DUOPAP mode, PDuo is the maximum pressure that is alternately applied to the previous baseline CPAP. Breathing rate is the number of PDuo applied per minute [12]. DUOPAP respiratory support increases mean airway pressure, tidal volume and minute ventilation and subsequently improves hypoxia and CO_2_ retention [12].

In this study, it is hypothesized that early use of NDUOPAP reduces the need for invasive respiratory support compared to NCPAP in preterm infants with respiratory distress syndrome.

## Methods

 This study was performed in a Neonatal Intensive Care Unit at Imam Khomeini Hospital of Ahvaz Jundishapur University of Medical Sciences in Ahvaz, Iran, during 2018–2019. Premature infants with gestational age of 28 to 34 weeks who had respiratory distress syndrome and their respiratory distress score based on the Silverman-Anderson table was 6 and 7 during the first 6 h of birth were enrolled [13–16].

Exclusion criteria included presence of major anomalies, airway anomaly, severe cardiovascular instability, respiratory distress secondary to severe asphyxia (Apgar score ≤ 3 at 1 and 5 min and umbilical cord blood pH < 7.1), parental discontent, gestational age less than 28 weeks, cyanotic heart disease, meconium aspiration syndrome, diaphragmatic hernia, invasive mechanical ventilation started from the beginning of hospitalization, pulmonary hemorrhage, lack of effective spontaneous breathing, metabolic disease during hospitalization and respiratory problems due to neuromuscular diseases and sepsis [12–16].

 All parents were required to complete and write an informed consent form before the neonates were enrolled in the study, according to the Ethics Committee of Jundishapur University of Medical Sciences (IR.AJUMS.REC.1397.365). Also, the present study was registered in the Iranian Clinical Trial Documentation Office on 10.9.2018 (IRCT: 2,018,082 1040847NI).

In this unmasked randomized trial, neonates were randomly divided into two groups of NDUOPAP and NCPAP.NDUOPAP group was considered group A and NCPAP group as group B. Based on the https://www.Sealedenvelope.com/simple-randomizer/V1/lists, the list was prepared. Six blocks were initially considered, including AABB, ABAB, ABBA, BABA, BAAB, BBAA and each block was assigned a code between 1 and 6. The statistical consultant randomly selected a number from 1 to 6 to create a random sequence and as a result, the infants were randomized into the two groups of A and B. Sample size was calculated by formula and according to the sample size of Zhou et al. [12] article, where the failure rates of non-invasive NDUOPAP and NCPAP treatment were 4.44 and 22.5 %, respectively, 67 patients were studied in each group. Due to the probability of at least 10 % sample attrition, 7 individuals were added to each group, resulting in a sample size of 148 (74 subjects per group). After birth, the necessary resuscitation procedures were performed by a trained resuscitation team and a senior physician assistant for all infants who weighed below 1500 g according to the NICU protocol and infants were transited to NICU in presence of a specialized NICU nurse under T-piece respiratory support (Fisher & paykel Healthcare, New Zealand) [16].

In the NICU, infants who were eligible for inclusion were randomly assigned to one of NDUOPAP or NCPAP groups. In infants of the DUOPAP group Fabian device (Acutronic, Switzerland, Infant flow driver) was used, which was connected to the infant via standard nasal tubes and injectors through a nasal prong. For neonates in this group, baseline parameters including PDuo (8 cm H_2_O) and CPAP (5 cm H_2_O), FIO_2_ 40 %, inhalation time of 0.5 s, and respiratory rate between 30 and 40 breaths per minute were adjusted. Based on clinical examination, arterial blood gas (ABG) and SPO_2_, device parameters were changed. The highest acceptable CPAP and PDuo levels were less than 8 cm H_2_O and 15 cm H_2_O, respectively, and the maximum FIO_2_ acceptable to continue treatment was 60 %. The goal of altering device setting was reaching SPO_2_ above 90 % in the right hand, PaO_2_ above 50 mmHg, PaCO_2_ less than 50 mmHg, pH above 7.25 and lack of respiratory distress on physical examination [12, 13].

In the NCPAP group, infants were subjected to Fabian device (Acutronic, Switzerland, Infant flow driver). The device was connected to the infant by standard injectors and tubes through the nasal prong. In the NCPAP group the initial parameters of the device were CPAP (5 cm H_2_O) and FIO_2_ 40 % and based on clinical examination, ABG and SPO_2_ changes of device parameters were performed. The highest acceptable CPAP level was less than or equal to 8 cm H_2_O and the maximum FIO_2_ acceptable to continue treatment was 60 %. The target was O_2_ saturation above 90 % in the right hand (PaO_2_ ≥ 50 cm H_2_ O, PaCO_2_ < 50 cm H_2_ O, and pH ≥ 7.25) and the absence of respiratory distress on physical examination [12, 13].

In both groups, based on existing therapeutic guides and under the direct supervision of the researcher, infants requiring FIO_2_ over 40 % with CPAP > 5 cm H_2_O to maintain O_2_saturation in the right hand between 90 and 95 %, 100 mg /kg surfactant (Survanta) were administered using the INSURE (Intubation, Surfactant and Extubation) method by a skilled practitioner who had been predetermined [17]. After INSURE, the infant received the same non-invasive ventilation used before INSURE.

A feeding tube was inserted to remove air from the baby’s stomach. O_2_ saturation was monitored and recorded by pulse oximeter and respiratory rate, heart rate was monitored continuously, and blood pressure every 2 h. In infants requiring a FIO_2_ greater than 40 % to maintain SPO_2_ within the acceptable range (90–95 %), surfactant was re-administered after 6 h after the last surfactant administration and as needed for a full course of treatment (maximum of 4 doses).

ABG was measured on admission (all subjects), in cases in need of intervention, one hour after the intervention as well as every 12 h thereafter, and before and after surfactant administration and the results were recorded in a special form. Based on the results an appropriate intervention was carried out when necessary [12, 16, 18, 19]. Occurrence of treatment failure as well as duration of intervention, pneumothorax, BPD, PDA, apnea, occurrence of death, IVH, duration of oxygen therapy, length of hospital stays and mean airway pressure were recorded every 6 h in each group. As decided, after improvement in patient’s condition and O_2_ saturation maintenance for 6 h, we went on to reduce the device settings, such that if in DUOPAP FIO_2_ was less than 30 % and CPAP and PDuo were less than or equal to 3 cm of water and 5 cm of water, respectively, and the infant was breathing continuously and ABG was normal for 24 h, the infant was disconnected from the apparatus and placed under oxyhood inhaling a mixture of air and oxygen with FIO_2_ 30–40 % and a flow of 5 to 10 L per minute depending on the size of the hood and patient’s O_2_ saturation [12].

In the CPAP group if the neonate was clinically stable (defined as respiratory rate lower than 60 per minute, no apnea and O_2_saturation > 90 % 0n right hand) parameters were reduced to: CPAP ≤ 3 cm H_2_O and FIO_2_ < 30 %. If neonate condition was stable for the preceding 24 h, the neonate was separated from CPAP [12].

All of the participants received antibiotics, caffeine as prophylaxis for apnea of prematurity and appropriate fluid and electrolyte solutions.

The primary outcome was the need for endotracheal intubation within the first 72 h of treatment. Treatment failure criteria included at least one of the following: pH < 7.2, PaCO_2_ > 60 mmHg, PaO2 < 50 mmHg with FIO_2_ > 60 %, CPAP > 8 cm H_2_O in NCPAP group and PDuo > 15 cm H_2_O, CPAP > 8 cm H_2_O, and FIO2 > 60 % in NDUOPAP group or worsening of the clinical condition (increased respiratory distress due to severe retraction) or prolonged apnea (stopping breathing for more than 20 s) or recurring apnea more than 2 times in 24 h with cyanosis and bradycardia (PR ≤ 100 / min) requiring ventilation with a bag and mask [12, 13, 20].

Secondary outcomes included duration of non-invasive ventilation, duration of oxygen therapy, duration of hospitalization, occurrence of IVH, pneumothorax, BPD, PDA, apnea, and death. All patients underwent echocardiogram within 48 h of birth and afterward if needed. Brain ultrasonography for diagnosing IVH was performed on the third and seventh days. Pneumothorax was diagnosed on the basis of chest x-ray and transillumination [11].

### Statistical analysis

In quantitative variables mean and standard deviation were used to describe the data in addition to median and interquartile range. Frequency and percentage were used to describe the data. Normality of the data was analyzed using Kolmogorov-Smirnov test and Q-Q chart. Data were analyzed using chi-square, Fisher’s exact test, t-test and Mann-Whitney test. Significance level was set at *P*-value less than 0.05. All analyses were performed using SPSS version 22.

## Results

**Fig. 1:**
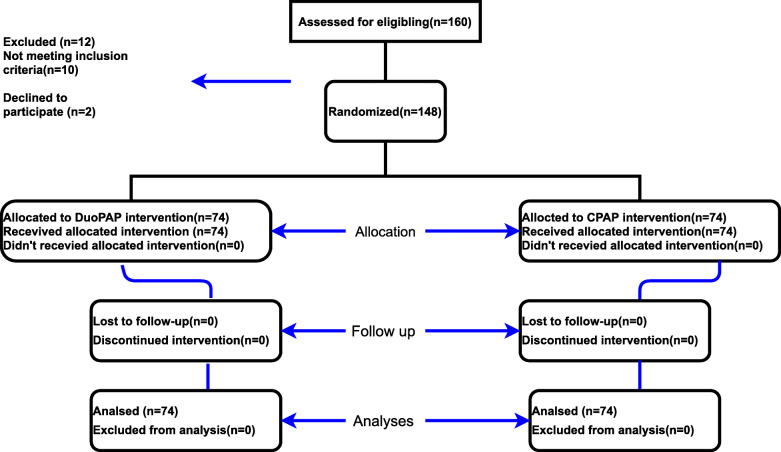
Consort. Transparent reporting of trials

According to Fig. 1, the study population consisted of 160 neonates born between 28 and 34 weeks of gestation with a diagnosis of RDS. A total of 12 neonates were excluded: 10 due to not meeting the inclusion criteria and 2 due to non-cooperation. Therefore, this study was performed on 148 infants, 74 treated with NCPAP and 74 treated with NDOUPAP.

The social and demographic characteristics of the infants are presented in Table 1. There were no significant differences in baseline characteristic. The level of arterial PCO_2_ one hour after inclusion in the NDUOPAP group (PaCO_2_:44.06 mmHg) was significantly lower than that of NCPAP (PaCO_2_:46.51 mmHg) and this difference was significant (*p* = 0.029). Arterial PO_2_ level was higher one hour after start of treatment in the NDUOPAP group (72.21 mmHg) than NCPAP (67.01 mmHg) (*p* < 0.001).

**Table 1:** Demographic and clinical Data in the study Groups

Characteristic	DUOPAP	CPAP	***P*** value
**Male, n (%)**	**40(54.1%)**	**32(43.2)**	**0.188**
**Female, n (%)**	**34(45.9%)**	**42(56.8)**	
**Cesarean delivery, n (%)**	**52(70.3%)**	**57(77%)**	**0.351**
**Vaginal delivery, n (%)**	**22(29.7%)**	**17(23%)**	
**Gestational age, weeks (mean ± SD)**	**31.32±1.53**	**31.13±1.77**	**0.618**
**Median (IQR)**	**31.55(2.1)**	**31.35(2.8)**	
**Body weight, gr (mean ±SD)**	**1415±233.15**	**1377.91±260.24**	**0.357**
**Antenatal steroids, n (%) First does**	**70(94.6%)**	**68(91.9%)**	**0.798**
**Antenatal steroids, n (%) second dose**	**2(2.7%)**	**3(4.1%)**	
**Without Antenatal steroids, n (%)**	**2(2.7%)**	**3(4.1%)**	
**APGAR 1 min (mean+ SD)**	**6.15±1.08**	**6.08±0.89**	**0.819**
**Median (IQR)**	**6(2)**	**6(2)**	
**APGAR 5 min (mean± SD)**	**7.69 ±0.79**	**7.70±0/81**	**0.928**
**Median (IQR)**	**8(1)**	**8(1)**	
**PPROM, n (%)**	**12(17.6%)**	**7(9.6%)**	**0.161**
**Age Mother, yrs. (mean ± SD)** **Mean IQR**	**30.08±7.44**	**29.24±6.59**	**0.470**
**Gestational age group, n (%)**			
**28-30 weeks**	**12 (16%)**	**19 (26%)**	**0.879**
**30-32 weeks**	**34 (46%)**	**23 (31%)**	
**32-34 weeks**	**28 (38%)**	**32 (43%)**	

*n* number, *SD* Standard Deviation, *IQR* Inter Quartile Range

There was no significant difference in the primary outcome of treatment failure during the first 72 h of birth between the NDUOPAP (3[4.1 %]) and NCPAP (6[8.1 %]) groups (*p* = 0.494); Table 2.

**Table 2:** Treatment effect and complication in study groups

Characteristic	NDUOPAP	NCPAP	***P*** value
**Failure in first 72 h, n (%)**	**3(4.1%)**	**6(8.1%)**	**0.494**
**Duration of Noninvasive Respiratory support (hr)** **(mean± SD)**	**39.18±18.14**	**50.12±23.83**	**0.004**
**Duration of oxygen therapy (hr)** **(mean± SD)**	**75.48±26.06**	**107.45±156.06**	**0.034**
**Duration of hospitalization (hr)** **Mean ± SD)**	**495.88±310.11**	**668.08±360.46**	**0.002**
**Pneumothorax, n median (IQR)**	**0(0)**	**2(2.7)**	**0.497**
**IVH, n (%)** **Grade I & II**	**3(4.1)**	**5(6.8)**	**0.719**
**PDA mild, n (%)**	**4(5.4%)**	**5(6.8%)**	**1**
**PDA moderate, n (%)**	**4(5.4)**	**3(4.1)**	**1**
**BPD, n (%)**	**0(0)**	**1(1.4)**	**0.319**
**Apnea, n (%)**	**1(1.4%)**	**4(5.4%)**	**0.366**
**Deaths, n (%)**	**2(2.7%)**	**5(6.8%)**	**0.442**
**Surfactant** **First dose, n (%)**	**31(41.9%)**	**39(52.7%)**	**0.042**
**Surfactant** **Secondary dose, n (%)**	**13(17.6%)**	**20(27%)**	**0.042**
**Surfactant** **3 dose, n (%)**	**2(2.7%)**	**1(1.4%)**	**0.042**

*h* hour, *PDA* Patent Ductus Arteriosus, *n* number, *BPD* Bronchopulmonary dysplasia, *IQR* Inter Quartile Range, *IVH* Intra Ventricular hemorrhage

The duration of non-invasive ventilation was shorter in the NDUOPAP group and this difference was significant (CPAP = 50.12 ± 23.83 h vs. DUOPAP = 39.18 ± 18.14 h; *p* = 0.004); Table 2.

The duration of oxygen therapy in the NDUOPAP group was shorter than that of NCPAP group (CPAP = 107.45 vs. DUOPAP = 75.48; *p* = 0.034;) Table 2.

Duration of hospitalization in the NDUOPAP group was shorter than that of NCPAP (CPAP = 668.08 h vs. DUOPAP = 495.88 h ; *p* = 0.02 ) Table 2.

Other outcomes including IVH, pneumothorax, BPD, PDA, apnea and death were not significantly different (*p* > 0.05); Table 2.

The mean airway pressure level in the NDUOPAP group was higher than that of the NCPAP group, but there was no significant difference between the two groups in terms of mean airway pressure at 72 h after birth.

## Discussion

In recent years, studies have focused on non-invasive ventilation techniques to reduce the need for mechanical ventilation and its associated pulmonary complications [4]. Since 1970, noninvasive ventilation has been widely used in infants with CPAP. Studies have shown that CPAP reduces the need for oxygen dependence, respiratory rate and the need for mechanical ventilation [21, 22].

However, non-invasive BIPAP ventilation during the respiratory cycle produces two levels of CPAP with frequency and duration as determined by the physician. Therefore, in theory BIPAP should perform better in alveolar deployment, functional residual capacity (FRC) and improvement respiratory function than CPAP. However, this has not yet been validated in clinical studies, and some studies have not yet demonstrated a clear link between BPD and non-invasive ventilation [23–26]. In this context, the present study aimed to compare the two non-invasive ventilation methods of NDUOPAP and NCPAP among 148 preterm infants with respiratory distress syndrome aged 28 to 34 weeks. Because infants weighing less than 1000 g and under 28 weeks of gestation are usually intubated and undergo mechanical ventilation, they were not included in this study [27, 28].

In the present study, the need for endotracheal intubation in the first 72 h of birth was not significantly different between the two groups (*p* = 0.494), which is similar to the results of Gao et al. [29], Aguiar et al. [30] and Victor S et al. [31] However, in the study of Zhou et al. [12] and Kong et al. [18], the need for endotracheal intubation was significantly lower in the NDUOPAP group than in the NCPAP group.

There was no statistically significant difference between the NDUOPAP and NCPAP groups in the present study. However, since the number of treatment failures in this study was three in the NDUOPAP group and six in the NCPAP group, despite the nonsignificant statistical difference between the two groups, this difference was clinically remarkable, which requires further investigations with larger sample sizes.

In this study, the amounts of PCO_2_ one hour after treatment in the NDUOPAP and NCPAP groups were 44.06 ± 4.11 mmHg and 46.51 ± 3.86 mmHg, respectively, which was statistically significant (*p* = 0.029), although this difference isn’t clinically considerable. This finding is consistent with the study of Zhou et al. [12] and Kong et al. [18]. The reason for this may be the improvement of the minute ventilation caused by the use of the NDUOPAP method [12].

In the present study, arterial blood PaO_2_ levels were also compared one hour after treatment in the NDUOPAP and NCPAP neonates, which were 72.21 ± 5.37 mmHg and 67.01 ± 6.57 mmHg, respectively, showing a significant difference between the two groups. This finding is also justified by the use of alveolar volume, flow and increased mean airway pressure (MAP) in patients treated with NDUOPAP [12, 32]. The findings of our study were similar to those of Zhou et al. [12] and Kong et al. [18].

In the present study, the mean duration of non-invasive ventilation between the NDUOPAP and NCPAP groups was 39.18 ± 18.14 h and 50.12 ± 23.83 h, respectively, which were significantly different (*p* = 0.004). This could be due to improved use of alveoli and accelerated production of surfactant. This significant difference may be the result of improved blood gas exchange in the neonate treated with NDUOPAP [11]. These findings were in agreement with the results of Lista et al. [19] and Arora et al. [32]. In the study of Zhou et al., the duration of non-invasive ventilation was similar in both NDUOPAP and NCPAP groups [12]. Also, in the study of GAO et al., no significant difference was found in the duration of noninvasive ventilation between the three groups of NCPAP, BIPAP and SBIPAP [29].

The duration of oxygen therapy in our study in the two groups NDUOPAP and NCPAP was 75.48 and 107.45 h, respectively, indicating a significant difference between the two groups (*p* = 0.034). This is also justified by improved alveolar deployment, improvement respiratory function and early respiratory system stability in patients treated under NDUOPAP treatment. These findings are consistent with those of Arora et al. [32] and Lista et al. [19].

The duration of hospitalization in the NDUOPAP and NCPAP groups was 495.88 and 668.08 h, respectively. There was a statistically significant difference between the two groups (*P* = 0.002). The results were consistent with those of Lista et al. [19] and Arora et al. [32]. This may be due to lower duration of non-invasive ventilation and oxygen therapy and earlier stabilization of the patient’s respiratory status.

The need for surfactant administration was also studied in both groups. The need for surfactant administration was significantly lower in NDUOPAP group (*p* = 0.042), which could be due to improved airway pressure and preventing alveolar collapse and thus reducing oxygen demand [33]. Alveolar stability during inhalation and exhalation may accelerate the production of surfactant and, on the other hand, achieve the ideal alveolar distribution of surfactant on alveolar surface. However, to prove this, separate studies are needed with larger sample sizes. In a study by Ricotta et al. in 2013, there was no significant difference between multiple doses of surfactant in the two groups of BiPAP and NIPPV [34].

In this study mortality was the same in both groups, probably because the number of treatment failure and prematurity complications were similar in both groups, which is similar to the studies of Arora et al. [32], Salvo et al. [35], and Gao et al. [29]. There was no significant difference between the two groups in terms of presence pneumothorax (*p* = 0.497), which is consistent with the results of Zhou et al. [12] and Lista et al. [19].

Bronchopulmonary dysplasia (BDP) was not significantly different between the two groups (*p* = 0.319). Many studies have investigated the incidence of PBD between different modes. Zhou et al. [12], Arora et al. [32] Rong et al. [36], and Lista et al. [19] obtained similar results.

The PDA (*P* = 1) and IVH (*P* = 0.1719) in both groups were similar, which was similar to findings of Zhou et al. [12]. Salvo’s results [35] showed no significant difference in IVH and PDA rates between the CPAP, BiPAP and NSIPPV groups. There was also no significant difference in IVH rate between the two groups of BiPAP and CPAP in the study of Gao et al. [29]. Similar results were found in the study of Lista et al. [19] regarding IVH.

There was no significant difference between the two groups in the rate of apnea in the present study (*P* = 0.366). This may be due to the low number of neonates with apnea and the lack of significance of this variable in the present study. Nursing reports on the severity of apnea are unreliable because existing devices cannot detect obstructive apnea or mixed apnea and can only record central apnea [37].

In our study, mean airway pressure was evaluated every 6 h in both modes. *P*-value up to 48 h was less than 0.001 and at 69 h it was *p* < 0.002. However, at 72 h, the *P*-value was equal to 0. 101, which may be due to separation of some patients from the device, thus decreasing the sample size (Table 3).

**Table 3:** Mean Airway Pressure difference during treatment in study groups

	MAP(CM/H2O) & Median		***P***-VALUE	Patient Number	
	NDUOPAP	NCPAP		NDUOPAP	NCPAP
At admit time	**6.89±0.76**	**5.29±0.47**	**<0.001**	**74**	**74**
	**6.85(1.30)**	**5.10(0.70)**			
After 6 Hour	**6.65±1.06**	**5.12 ±0.63**	**<0.001**	**74**	**73**
	**6.50(1.13)**	**5(0.4)**			
After 12 Hour	**6.07± 1.03**	**4.75± 0.49**	**<0.001**	**72**	**71**
	**6.25(1.17)**	**4.90(0.50)**			
After 18 Hour	**5.63± 1.41**	**4.46± 0.72**	**<0.001**	**72**	**71**
	**5.95(2.40)**	**4.80(1)**			
After 24 Hour	**5.11± 1.20**	**4.28±0.85**	**<0.001**	**67**	**71**
	**5.30(2.2)**	**4.70(1.60)**			
After 30 Hour	**5.17± 1.12**	**4.65± 3.74**	**<0.001**	**48**	**58**
	**5.30(2.22)**	**4.10(1)**			
After 36 Hour	**4.77± 1.07**	**3.96± 0.66**	**<0.001**	**43**	**52**
	**4.2(1.5)**	**4(0.9)**			
After 42 Hour	**4.64± 1.02**	**3.69± 0.71**	**<0.001**	**40**	**27**
	**4.1(1.55)**	**3.6(o.9)**			
After 48 Hour	**4.67± 1.10**	**3.64± 0.76**	**<0.001**	**26**	**42**
	**4.10(1.65)**	**3.25(1.10)**			
After 60 Hour	**4.43± 0.92**	**3.49± 0.61**	**0.002**	**10**	**25**
	**4.20(1.35)**	**3.20(1)**			
After 72 Hour	**3.88± 0.53**	**3.39± 0.45**	**0.101**	**5**	**17**
	**4.10(085)**	**3.20(0.90)**			

### Limitations

Limitations of this study include limited sample size and exclusion of infants with gestational age less than 28 weeks in this study. A multicenter study is needed to further validate these findings.

## Conclusions

In this study, NDUOPAP was compared to NCPAP and did not decrease the need for mechanical ventilation in the first 72 h of birth, but the duration of non-invasive ventilation, duration of oxygen requirement, and duration of hospitalization in the NDUOPAP group were lower. However, further studies are needed to evaluate the potential benefits of non-invasive ventilation, especially for vulnerable preterm infants or low Apgar infants.

## Data Availability

The datasets generated and analyzed during the current study are available from the corresponding author on reasonable request.

## Abbreviations

ABG: Arterial blood gas
BIPAP: Bilevel positive airway pressure
BPD: Bronchopulmonary dysplasia
DUOPAP: Duo positive airway pressure
HFNC: High flow nasal cannula
IVH: Intraventricular hemorrhage
MAP: Mean airway pressure
NCPAP: Nasal continuous positive airway pressure
NHFO: Nasal high frequency oscillation
NIPPV: Nasal intermittent positive ventilation
PDA: Patent ductus arteriosus
RDS: Respiratory distress syndrome
